# Acute Effect of Submaximal Exercise on Respiratory System Impedance in Healthy Adults

**DOI:** 10.3390/jfmk10040403

**Published:** 2025-10-17

**Authors:** Thales Henrique do Carmo Furquim, Daniele Oliveira dos Santos, Jéssica Perossi, Fernanda Cristina Lima, Janne Marques Silveira, Ada Clarice Gastaldi

**Affiliations:** 1 Department of Health Sciences, Program in Rehabilitation and Functional Performance, Ribeirão Preto Medical School, University of São Paulo, Ribeirão Preto 14049-900, Brazil; thales.furquim@usp.br (T.H.d.C.F.); daniolivfisio@gmail.com (D.O.d.S.); jessicaperossi@gmail.com (J.P.); jannems@usp.br (J.M.S.); 2 Department of Health Science, University of Gurupi, Tocantins 77403-080, Brazil

**Keywords:** physiotherapy, airway resistance, pulmonary function test, six-minute walk test, respiratory system, healthy volunteers

## Abstract

**Background:** Previous studies have experimentally investigated and simulated the effects of dynamic hyperinflation on respiratory system resistance variables in populations with different respiratory diseases. However, the acute effects of exercise on these parameters in healthy young adults are unknown; therefore, the objective of this study was to investigate the effects of a submaximal exercise test on the resistance of the respiratory system in healthy adult individuals. **Methods:** This is an observational study conducted with healthy adults. Fifty healthy volunteers were recruited, of both sexes, over the age of 18, with no previous uncontrolled respiratory diseases and normal spirometry, with an average age of 32.2 ± 11.6 years old and an average BMI of 24.3 ± 3.87 kg/m^2^. The participants underwent an anamnesis, the Impulse Oscillometry Test (IOS) with the variables R5, R20, X5, Fres and AX, followed by the six-minute walk test (6MWT), another IOS measure, immediately after the 6MWT, finishing with a spirometry test. A paired *t*-test was used for the analyses. **Results:** Our results showed that the volunteers exhibited a decrease after the 6MWT in the oscillometry parameters R5 (0.35 to 0.32, *p* = 0.0001), R20 (0.33 to 0.30, *p* < 0.0001), X5 (−0.13 to −0.10, *p <* 0.0001), Fres (13.07 to 11.70, *p* = 0.0042) and AX (0.48 to 034, *p* = 0.01). In addition, the volunteers walked an average of 623.34 ± 42.6 m in the 6MWT, which represents around 96.6% of what was predicted for this group. **Conclusions:** Submaximal exercise, as assessed by the 6MWT, acutely reduces respiratory impedance in healthy individuals, which can facilitate moderate-intensity physical activity, preventing sedentary behavior.

## 1. Introduction

The main function of the respiratory system is to supply the body with oxygen (O_2_) and remove carbon dioxide (CO_2_), the result of cellular metabolism [1]. The lungs and chest wall, therefore, are specialized structures that facilitate the transport of gases by means of their elastic properties, with the lungs tending to collapse and the chest wall tending to expand, based on the physiological concepts of compliance and elasticity. The properties of these two structures remain in balance; however, situations such as lung hyperinflation can cause biomechanical disadvantages for the diaphragm muscle and impair breathing efficiency [2,3].

In addition, other structures that make up the respiratory system, such as the bronchi, influence airway resistance, and, physiologically, the smaller the radius of these structures, the greater the resistance. In practical terms, it is easier for airflow to pass through a larger-diameter tube than a smaller-diameter one [1,4].

An increase in respiratory rate and a reduction in tidal volume, which occur in response to the increase in ventilatory demand during exercise, can favor dynamic hyperinflation. Exercise-induced dynamic lung hyperinflation and the recruitment of the deep abdominal muscles during exhalation can lead to a reduction in the inspiratory capacity in patients with Chronic Obstructive Pulmonary Disease (COPD), which contributes to ventilatory overload and a progressive increase in discomfort [5].

The literature describes experimental situations developed to simulate the increase in ventilatory demand observed during physical exercise, using, for example, voluntary control of respiratory rate and the induction of pulmonary hyperinflation [6,7]. Our research group has already explored this model in a previous study, in which we simulated the ventilatory conditions of physical exercise by means of controlled maneuvers at rest, with a focus on evaluating the resistance parameters of the respiratory system using the Impulse Oscillometry System (IOS) [8].

However, this approach was unable to reproduce the complete physiological adaptations that occur during exercise situations, such as the simultaneous increase in ventilatory and metabolic demand, but the acute effects of exercise in the impedance of the respiratory system are still underexplored. The effect of exercise on respiratory resistance, although discussed in respiratory diseases [9,10], has been little explored in healthy adult individuals; exploring this effect would allow us to understand the physiological and pulmonary mechanics in individuals without previous respiratory disease, expanding our knowledge and improving the applicability of exercise interventions in other populations.

Our hypothesis is that physical exercise could alter the resistance of the respiratory system in healthy adults, although this has already been previously demonstrated by our group in a simulation with individuals with chronic respiratory diseases [8]. To test this hypothesis, we assessed the acute impact of submaximal exercise on respiratory impedance assessed via impulse oscillometry in healthy adults.

## 2. Material and Methods

### 2.1. Study Design and Ethical Approval

This is a cross-sectional observational study with a convenience sample that was submitted to the Research Ethics Committee of the Hospital das Clínicas of the Ribeirão Preto Medical School of the University of São Paulo (HCFMRP-USP), and was approved under CAAE number 67006923.5.0000.5440. The study followed the standards and recommendations of Strengthening the Reporting of Observational Studies in Epidemiology (STROBE).

### 2.2. Study Location

Data was collected at the Respiratory Assessment Laboratory (LAR) in the Department of Health Sciences at the Ribeirão Preto Medical School of the University of São Paulo (FMRP-USP).

### 2.3. Eligibility Criteria

The inclusion criteria included volunteers who were at least 18 years old, with all genders considered, all of whom self-reported that they did not have COVID-19 or another diagnosed respiratory disease and normal spirometry results. And the exclusion criteria included volunteers with impaired understanding of the test or incapacity to undergo the testing, who were pregnant, and who had unmanaged breathing issues.

### 2.4. Recruitment and Selection of Participants

To recruit volunteers, social media such as Instagram was used, as well as WhatsApp, websites, newspapers, pamphlets and television, the latter through an interview with G1 (Globo News Portal—local television news program), in the Ribeirão Preto region. When candidates were identified, the inclusion and exclusion criteria were checked and invitations to take part in the research were sent virtually; after agreeing to take part in person on the day of the assessment, the volunteers were given the informed consent form (ICF) to sign before data collection began.

### 2.5. Evaluation

**Figure 1:**
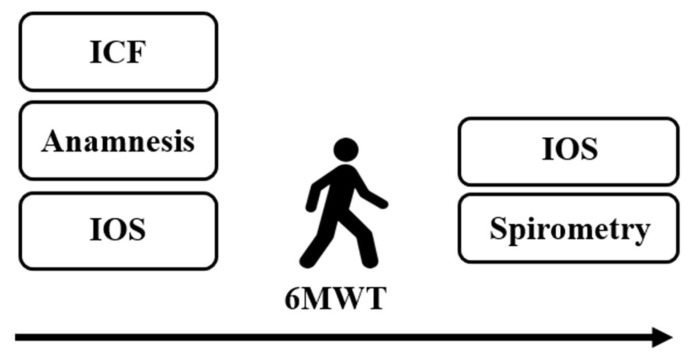
Flowchart of the assessment day. **ICF**: informed consent form; **IOS:** Impulse Oscillometry System; **6MWT:** six-minute walk test.

Initially, anamnesis was conducted with the volunteers to collect sociodemographic and anthropometric data, as well as personal history and health history. Next, the participants underwent a previous-pulmonary-function test to assess the impedance of the respiratory system using IOS; then they performed the 6MWT and repeated the IOS assessment immediately after (less than a minute) completing the 6MWT, ending the test with a spirometry exam (Figure 1).

### 2.6. Impulse Oscillometry System—IOS

Jaeger IOS equipment (Jaeger, Wurzburg, Germany)—acquired under FAPESP process 2013/26433-0—was used to assess the resistance of the respiratory system. The test was conducted as described by Brashier and Salvi, 2015, and Oosteeven and cols, 2003 [11,12]. The volunteer was seated on a height-adjustable chair to explain the test and positioned with their knees at 90º and feet on the floor, without crossing their legs; a nose clip and bacteriological filter were used during the test, as well as a mouthpiece with a tongue depressor. The participant was instructed to breathe through their mouth at tidal volume (VT) with their hands resting firmly on their cheeks, and each measurement lasted 30 s, with 3 measurements being taken by the device, and to ensure test reproducibility, if there was a variation greater than 15% between the recorded curves, the measurement was excluded. The reference equation chosen was that of Vogel and Smidt from 1994 [13], suitable for subjects aged 18 to 69, to calculate the predicted values for each individual.

The Vogel and Smidt equation, 1994, is as follows:

*R* = *R_Io_* + *dR*/*d* [*V*]’ × [*V*]’ + *dR*/*dV* × (*V* − *V_Io_*)

*X* = *X_Io_* + *dX*/*d* [*V*]’ × [*V*]’ + *dX*/*dV* × (*V* − *V_Io_*)

where **R** = respiratory resistance; **X** = respiratory reactance; **R_Io_** = inspiratory resistance at zero flow; **X_Io_** = inspiratory reactance at zero flow; **dR/dV** = volume gradients of resistance; **dX/dV** = volume gradients of the reactance; **V** = volume; and **V_Io_** = inspiratory volume at zero flow.

The equipment involved components such as a loudspeaker, a Y-adapter and a pneumotachograph. This speaker is responsible for emitting sound waves at different frequencies, given in Hertz (Hz). Lower frequencies travel longer distances, reflecting the total resistance of the respiratory system (R5), while higher frequencies travel shorter distances in the respiratory system, reflecting the resistance of larger-caliber airways (R20). The difference between one parameter and the other is what we call R5-R20 and reflects the resistance of smaller-caliber airways. Furthermore, IOS can provide relevant information about the elastic properties of the lung and is called the reactance of the respiratory system, represented by X5, and involves concepts of inertance and capacitance. The resonance frequency (Fres) represents the moment when the capacitive and inertial pressures are equal. The reactance area (AX) can be influenced by the reactance parameters, which increase or decrease their values.

### 2.7. Six-Minute Walk Test—6MWT

The aim of this test was to assess the volunteers’ functional exercise capacity, and it was conducted in accordance with the standards specified in the 2002 American Thoracic Society (ATS) document *Guidelines for the six-minute walk test* [14]. In a flat 30 m corridor, the participant was seated in a chair for around 10 min before the test began, and during this time information was collected on vital signs such as heart rate (HR), respiratory rate (RR), peripheral oxygen saturation (SpO_2_)—using a digital pulse oximeter (Geratherm oxy control, Germany) which the patient carried with them throughout the test—blood pressure (BP), and perception of dyspnea and fatigue in the lower limbs (lower limbs) using the Modified Borg Scale (0–10). Next, the volunteer was instructed to walk as fast as possible, without running, circling a chair that marked the beginning and end of the route, for a period of 6 min. Every minute, as highlighted in the ATS guidelines [14], the therapist conducting the test repeated standard phrases to encourage the volunteer to keep up the walking pace—the guidelines advise the following: “after the first minute, say the following to the patient (in a consistent tone): ‘You are doing well. You have 5 min left’. When the timer shows 4 min left, say the following to the patient: ‘Keep it up. You have 4 min left’. When the timer shows 3 min left, say the following to the patient: ‘You are doing very well. You are halfway there’. When the timer shows 2 min left, say the following to the patient: ‘Keep it up. Only 2 min left’. When the timer shows only 1 min left, say the following to the patient: ‘You are doing very well. Only 1 min left’”. No other verbal or other kind of incentive was offered to the participant; halfway through the test, data on HR, SpO_2_ and the perception of dyspnea and fatigue in the lower limbs were collected. At the end of the test, the volunteer was taken to the laboratory for impulse oscillometry to be carried out again, with their vital signs collected at the 6th, 9th and 12th min. The reference equation chosen to calculate the predicted distance was that of Britto et al., 2013 [15], which is suitable for the Brazilian population.

### 2.8. Spirometry

The test was performed following the 2019 update of the American Thoracic Society (ATS) and European Respiratory Society (ERS) [16] guidelines for standardizing spirometry and was performed on the same device as the impulse oscillometry described above. The volunteer was seated in a height-adjustable chair, and the spirometry maneuver performed was forced vital capacity (FVC), so the therapist instructed the participant to breathe at tidal volume for about 5 incursions; then the volunteer was instructed to exhale fully to residual volume (RV) and then inhale fully to total lung capacity (TLC). The final instruction was for the individual to exhale explosively and quickly to RV and to continue exhaling for at least 6 s before inhaling calmly again. At least three maneuvers were performed until there were three acceptable curves and two reproducible ones, with at least one minute’s rest between maneuvers. A nose clip and a bacteriological filter were used to carry out the test The reference equation chosen to calculate the predicted values for each individual was that of Pereira et al., 2007 [17].

### 2.9. Statistical Analysis

For descriptive statistical analysis, the volunteers’ data were tabulated in an Excel 2007 spreadsheet and presented as means and standard deviations. The data were analyzed using GraphPad Prism statistical software, version 10.2.0 (GraphPad Software, Inc.; Boston, MA, USA). Data distribution was tested using the Shapiro–Wilk test. For analyses between pre- and post-6MWT moments, the Student’s paired *t*-test or Mann–Whitney U test was used. The significance level was set at 5%.

## 3. Results

We analyzed 50 healthy individuals with no previous diagnosis of COVID-19 and no previous known respiratory disease. The characteristics of the participants at baseline are shown in Table 1. The sample consisted of 21 men and 29 women, all eutrophic adults (BMI 24.3 ± 3.87 Kg/m^2^), with an average age of around 32 years ± 11.60 and physically active (only 10 individuals reported not practicing physical activity regularly). The spirometry variables are presented in Table 1, with all of the lung function parameter values above the lower limit of 80%, demonstrating that the sample of healthy individuals had preserved lung function (%FVC 98.85; %FEV_1_ 99.24; %FEV_1_/FVC 99.61). It is worth noting that nine of the fifty participants did not perform the spirometry test satisfactorily, resulting in non-reproducible and unacceptable curves, which were excluded from the final analysis just for this variable, as indicated in Table 1 for spirometry parameters (n = 41).

Table 2 shows the performance of the volunteers during the 6-minute walk test, where the average distance covered during the test was approximately 623 m, which represents around 96% of the predicted distance for this sample, according to the equation by Britto et al., 2013 (15), indicating that the individuals performed the submaximal stress test proposed in our protocol satisfactorily.

**Figure 2:**
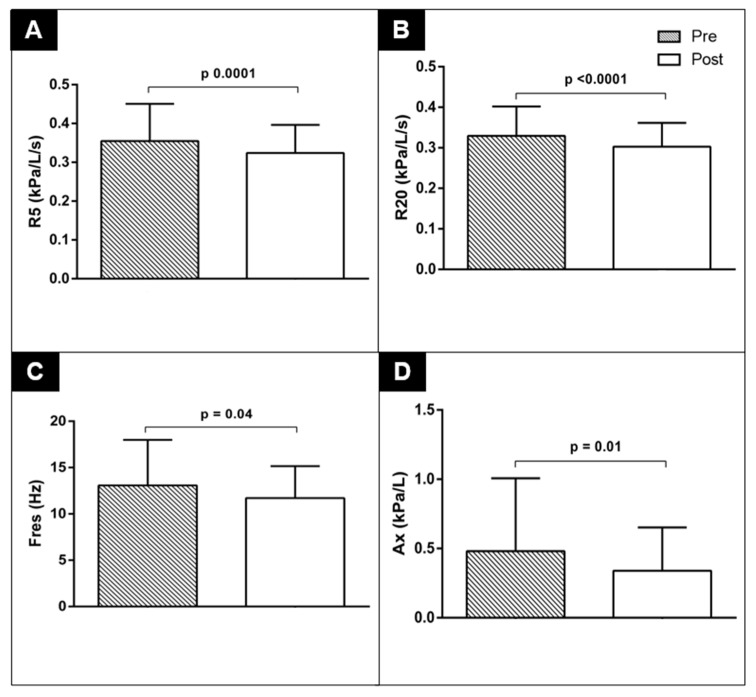
Comparison of pre- and post-TC6 IOS parameters. (**A**) **R5** (total resistance of the respiratory system); (**B**) **R20** (resistance of larger-caliber airways); (**C**) **Fres** (resonant frequency); (**D**) **Ax** (reactance area).

Our results in Table 2 also show that the volunteers exhibited a decrease after the 6MWT in the oscillometry parameters R5 (*p* = 0.0001), %R5 (*p* < 0.0001), R20 (*p* < 0.0001) and %R20 (*p* < 0.0001), X5 (*p* < 0.0001), Fres (*p* = 0.0042) and AX (*p* = 0.01). These differences can be found in Figure 2, and all of the results are shown in Table 2.

## 4. Discussion

This is the first study to evaluate the acute effect of submaximal exercise on the impedance of the respiratory system in healthy adult volunteers, and a reduction in resistance and reactance parameters was observed after the 6 min walk test. The spirometry of these volunteers revealed that their lung function was preserved, which reinforces the appropriate lung capacity of the participants.. The participants’ performance in the 6MWT was satisfactory, with more than 95% of the expected distance covered; this validates the reproduction of the submaximal stress test performed by these individuals with an assessment of the resistance of the respiratory system immediately after the end of the test, which reflects what happened during the exercise, which demonstrates the originality of this study.

After the 6 min walk test, there was a decrease in respiratory system resistance parameters in terms of the absolute values and as a percentage of predicted values (R5, %R5, R20, %R20), reactance (X5), Fres and reactance area (AX), which is particularly interesting as it shows that even in young, healthy adults, with normal previous values, submaximal physical exertion is capable of causing detectable changes in respiratory mechanics; demonstrating the sensitivity of impulse oscillometry in capturing exercise-induced physiological adaptations; it stands out as a lung assessment test that is quick and comfortable to perform, as it is carried out using tidal volume and therefore requires no effort from the volunteer, providing compartmentalized information on the respiratory system [11,18].

In contrast to our results, a study that assessed the resistance of the respiratory system using the IOS in schoolchildren aged between 6 and 14 after the 6MWT showed an increase in R5 and R20 after the test [19], unlike our findings, which showed a decrease in these parameters. This divergence may be related to natural physiological differences between the groups due to their different age groups. Children’s airways are narrower and more prone to collapse, and autonomic control is less matured in children, which can predispose them to exercise-induced bronchospasm, even in individuals without diagnosed respiratory disease, decreasing the radius of the airway and increasing its resistance [1,19]. In addition, the mechanical behavior of the airways in the face of exertion can vary throughout development, which reinforces the importance of considering the age group when interpreting the effects of exercise on respiratory mechanics [20,21].

Our volunteers were subjected to a submaximal exercise test, a situation that produced an increase in respiratory rate, promoting dynamic pulmonary hyperinflation, which is generally associated with a mechanically disadvantageous position for the respiratory muscles, causing increased respiratory work and limiting exercise [22]. However, the increase in lung volume can contribute to a decrease in airway resistance, as observed in our study, with a reduction in R5 and R20, due to its radial traction, with a consequent increase in caliber with the increase in lung volume, a mechanism by which the resistance parameters possibly decreased in our study. Additionally, Anderson and Daviskas (2000) highlight that exercise-induced hyperventilation and an increase in lung volume can modify airway tone [23]. In another study published by our group, ventilatory simulation using respiratory rate control and voluntary hyperinflation made it possible to assess the effects on airway resistance under artificial conditions, but was unable to fully reproduce the complex interaction between ventilatory and metabolic demand present during real physical exercise [8]. Thus, this study represents an important methodological advancement, making it possible to directly observe the effects of submaximal effort on respiratory mechanics in healthy individuals.

Situations of low lung compliance such as fibrosis or hyperinflation can lead to more negative reactance values (X5), and this parameter provides information that reflects what happens in more peripheral airways, so the more negative X5, the greater the hindrance of airflow, especially in more distal airways [23]. The healthy subjects showed a reduction in X5 (more positive values) after the 6MWT, which could be considered beneficial, since exercise may have improved the elasticity of their respiratory system, making the lungs “less rigid” and favorable for air entry [24,25,26], which could reduce exercise intolerance, according to our findings.

Resonance frequency tends to increase in both obstructive and restrictive diseases [27], and normal values for adults are between 7 and 12 Hz [28,29]. There was a decrease in Fres after the 6MWT in our sample, which is consistent with the decrease in some impedance parameters of the respiratory system, such as AX, which, when increased, can indicate peripheral airway obstruction [18].

The practice of physical activity and exercise is recommended for patients with chronic respiratory diseases because it is associated with improvement parameters in these patients. Thus, these results in healthy subjects suggest that, if the same effect could be observed in patients with chronic respiratory diseases, strategies to control respiratory rate and lung volume may be beneficial during exercise. In healthy subjects, the reduction in Fres after the 6 min walk test is consistent with the reduction in resistance parameters and may indicate the involvement of large (R20) and small (X5 and AX) airways in the reduction mechanism. In view of this, our findings on the acute effect of exercise on respiratory system resistance in a healthy population may help patients adhere to pulmonary rehabilitation programs, as it may contribute to a reduced sensation of dyspnea during exercise.

It is not possible to determine whether the reduction in respiratory system resistance parameters observed after the 6 min walk test represents an acute and transient effect of submaximal exercise or whether it could be maintained for longer periods or even enhanced by regular physical activity, and whether this varies according to the type of exercise (aerobic, muscle strength training). Furthermore, we did not evaluate different intensities or durations of exercise, which limits our understanding of how longer or more intense stimuli could impact respiratory mechanics.

### Limitations

This study presents have some limitations. We evaluated only on the immediate effects of submaximal exercise, due to the cross-sectional nature of our research. Nine out of fifty participants were excluded from the spirometry analysis, which may have affected the robustness of the conclusions. Also, we use only the 6MWT as a submaximal exercise. For future research, it is recommended to include a follow-up assessment, to explore different exercise intensities and durations; in addition, future studies should aim for randomized or stratified sampling to improve generalizability.

## 5. Conclusions

Submaximal exercise, here standardized by the 6MWT, is capable of acutely reducing respiratory system impedance in healthy individuals, which can facilitate moderate-intensity physical activity, preventing sedentary behavior. It is important to assess whether the same results can be obtained in patients with various chronic respiratory diseases to guide respiratory physiotherapists when proposing respiratory exercises and strategies for the treatment of patients with these conditions, as this could optimize clinical practice and assist airflow limitation in those obstructive pulmonary diseases.

## Figures and Tables

**Table 1:** Characteristics of the volunteers.

Anthropometric Variables (n = 50)	
**Male**	21
**Female**	29
**Sedentary**	10/50
**Age (Years)**	**Mean and standard deviation**
	32.22 ± 11.60
**Weight (Kg)**	69.62 ± 14.18
**Height (m)**	1.68 ± 0.09
**BMI (Kg/m^2^)**	24.3 ± 3.87
**Spirometry parameters (n = 41)**	
**CVF (L)**	4.32 ± 1.20
**%CVF**	98.85 ± 13.47
**VEF_1_ (L)**	3.62 ± 0.99
**%VEF_1_**	99.24 ± 14.41
**VEF_1_/CVF**	83.87 ± 6.73
**%VEF_1_/CVF**	99.61 ± 6.89
**FEF_25–75%_ (L/min)**	3.94 ± 1.32
**%FEF_25–75%_**	98.15 ± 27.21

Values are mean ± standard deviation, mean ± SD.

**Table 2:** Impulse oscillometry parameters before and after the 6MWT and participants’ performance in the test.

Parameters	Pre-6MWT	Post-6MWT	*p*
**R5 (kPa/L/s)**	0.35 ± 0.10	0.32 ± 0.07	**0.0001**
**%R5 (kPa/L/s)**	113.70 ± 30.26	103.36 ± 21.68	**<0.0001**
**R20 (kPa/L/s)**	0.33 ± 0.07	0.30 ± 0.06	**<0.0001**
**%R20 (kPa/L/s)**	126.24 ± 26.30	115.98 ± 21.38	**<0.0001**
**R5-R20 (kPa/L/s)**	0.03 ± 0.04	0.02 ± 0.04	0.19
**%R5-R20 (kPa/L/s)**	55.50 ± 90.28	43.33 ± 79.88	0.07
**X5 (kPa/L/s)**	−0.13 ± 0.05	−0.10 ± 0.04	**<0.0001**
**Fres (Hz)**	13.07 ± 4.93	11.70 ± 3.45	**0.0042**
**AX (kPa/L)**	0.48 ± 0.53	0.34 ± 0.31	**0.01**
**Distance covered in the 6MWT (m)**	623.34 ± 42.6		
**%prev TC6**	96.6 ± 7.4		

Values are mean ± standard deviation, mean ± SD. **m** (meters); **%prev** (percentage of predicted); **R5**, resistance at 5 Hz, which means total respiratory resistance; **R20**, resistance at 20 Hz, which means resistance of larger-caliber airways; **R5–R20**, resistance of smaller-caliber airways; **X5**, total reactance of the respiratory system; **Fres**, resonant frequency; **AX**, reactance area; **kPa/L/s,** Kilopascals per liter per second; **kPa/L**, Kilopascals per liter.

## Data Availability

The original data presented in the study are openly available in the University of São Paulo Repository (USP Digital Repository) at https://uspdigital.usp.br/repositorio/detalhe-dados.jsp?id-dados=670.

## Abbreviations

The following abbreviations are used in this manuscript:

O_2_	Oxygen
CO_2_	Carbon dioxide
COPD	Chronic Obstructive Pulmonary Disease
IOS	Impulse Oscillometry System
6MWT	6-min walk test
STROBE	Strengthening the Reporting of Observational Studies in Epidemiology
TV	Tidal volume
Hz	Hertz
R5	Total resistance of the respiratory system
R20	Resistance of larger-caliber airways
R5-R20	Resistance of smaller-caliber airways
X5	Total reactance of the respiratory system
Fres	Resonant frequency
AX	Reactance area
HR	Heart rate
RR	Respiratory rate
SpO_2_	Peripheral oxygen saturation
BP	Blood pressure
ATS	American Thoracic Society
ERS	European Respiratory Society
FVC	Forced vital capacity
RV	Residual volume
TLC	Total lung capacity
BMI	Body mass index
FEV_1_	Force expiratory volume in the first second
kPa/L/s	Kilopascals per liter per second
kPa/L	Kilopascals per liter

## References

[1] West J.B. (2013). Fisiologia Respiratória: Princípios Básicos.

[2] Fishman A.P. (1985). Handbook of Physiology. The Respiratory System. Circulation and Nonrespiratory Functions.

[3] Hlastala M.P. (2001). Physiology of Respiration.

[4] Leff A.R. (1993). Respiratory Physiology: Basics and Applications.

[5] Vogiatzis I., Zakynthinos S., Prakash Y.S. (2012). Factors Limiting Exercise Tolerance in Chronic Lung Diseases. Organizador. Comprehensive Physiology [Internet].

[6] Albuquerque C.G.D., Andrade F.M.D.D., Rocha M.A.D.A., Oliveira A.F.F.D., Ladosky W., Victor E.G., Rizzo J.Â. (2015). Determining respiratory system resistance reactance by impulse oscillometry in obese individuals. J. Bras. Pneumol..

[7] Oppenheimer B.W., Berger K.I., Segal L.N., Stabile A., Coles K.D., Parikh M., Goldring R.M. (2014). Airway Dysfunction in Obesity: Response to Voluntary Restoration of End Expiratory Lung Volume. PLoS ONE.

[8] Moroli R.G., Santos D.O.D., Souza H.C.D.D., Perossi L., Ribeiro M.A., Perossi J., Baddini-Martinez J.A., Gastaldi A.C. (2021). Effects of Controlled Voluntary Increase in the Ventilatory Demand on Respiratory System Resistance in Healthy and Non-Cystic Fibrosis Bronchiectasis Subjects: A Cross-Sectional Study. Arch. Bronconeumol..

[9] Malmberg L.P., Mäkelä M.J., Mattila P.S., Hammarén-Malmi S., Pelkonen A.S. (2008). Exercise-induced changes in respiratory impedance in young wheezy children and nonatopic controls. Pediatr. Pulmonol..

[10] Tiller N.B., Cao M., Lin F., Yuan W., Wang C.Y., Abbasi A., Calmelat R., Soriano A., Rossiter H.B., Casaburi R. (2021). Dynamic airway function during exercise in COPD assessed via impulse oscillometry before and after inhaled bronchodilators. J. Appl. Physiol..

[11] Brashier B., Salvi S. (2015). Measuring lung function using sound waves: Role of the forced oscillation technique and impulse oscillometry system. Breathe.

[12] Oostveen E., MacLeod D., Lorino H., Farre R., Hantos Z., Desager K., Marchal F. (2003). The forced oscillation technique in clinical practice: Methodology, recommendations and future developments. Eur. Respir. J..

[13] Vogel J., Smidt U. (1994). Impulse Oscillometry. Analysis of Lung Mechanics in General Practice and the Clinic, Epidemiology and Experimental Research.

[14] (2002). ATS Statement: Guidelines for the six-minute walk test. Am. J. Respir. Crit. Care Med..

[15] Britto R.R., Probst V.S., Andrade A.F.D.D., Samora G.A.R., Hernandes N.A., Marinho P.E.M., Karsten M., Pitta F., Parreira V.F. (2013). Reference equations for the six-minute walk distance based on a Brazilian multicenter study. Braz. J. Phys. Ther..

[16] Graham B.L., Steenbruggen I., Miller M.R., Barjaktarevic I.Z., Cooper B.G., Hall G.L., Hallstrand T.S., Kaminsky D.A., McCarthy K., McCormack M.C. (2019). Standardization of Spirometry 2019 Update. An Official American Thoracic Society and European Respiratory Society Technical Statement. Am. J. Respir. Crit. Care Med..

[17] Pereira C.A.D.C., Sato T., Rodrigues S.C. (2007). Novos valores de referência para espirometria forçada em brasileiros adultos de raça branca. J. Bras. Pneumol..

[18] Porojan-Suppini N., Fira-Mladinescu O., Marc M., Tudorache E., Oancea C. (2020). Lung Function Assessment by Impulse Oscillometry in Adults. TCRM.

[19] Assumpção M.S.D., Ribeiro J.D., Wamosy R.M.G., Parazzi P.L.F., Schivinski C.I.S. (2018). Oscilometria de Impulso e Espirometria em Escolares Submetidos ao Teste de Caminhada de Seis Minutos. Rev. Paul. Pediatr..

[20] Parshall M.B., Schwartzstein R.M., Adams L., Banzett R.B., Manning H.L., Bourbeau J., Calverley P.M., Gift A.G., Harver A., Lareau S.C. (2012). An Official American Thoracic Society Statement: Update on the Mechanisms, Assessment, and Management of Dyspnea. Am. J. Respir. Crit. Care Med..

[21] Gotshall R.W. (2006). Airway Response during Exercise and Hyperpnoea in Non-Asthmatic and Asthmatic Individuals. Sports Med..

[22] O’Donnell D.E., Revill S.M., Webb K.A. (2001). Dynamic Hyperinflation and Exercise Intolerance in Chronic Obstructive Pulmonary Disease. Am. J. Respir. Crit. Care Med..

[23] Anderson S.D., Daviskas E. (2000). The mechanism of exercise-induced asthma is …. J. Allergy Clin. Immunol..

[24] Van Noord J., Clement J., Cauberghs M., Mertens I., Van De Woestijne K., Demedts M. (1989). Total respiratory resistance and reactance in patients with diffuse interstitial lung disease. Eur. Respir. J..

[25] Sokai R., Ito S., Iwano S., Uchida A., Aso H., Kondo M., Ishiguro N., Kojima T., Hasegawa Y. (2016). Respiratory mechanics measured by forced oscillation technique in rheumatoid arthritis-related pulmonary abnormalities: Frequency-dependence, heterogeneity and effects of smoking. SpringerPlus.

[26] Kaminsky D.A., Simpson S.J., Berger K.I., Calverley P., De Melo P.L., Dandurand R., Dellacà R.L., Farah C.S., Farré R., Hall G.L. (2022). Clinical significance and applications of oscillometry. Eur. Respir. Rev..

[27] Michaelson E.D., Grassman E.D., Peters W.R. (1975). Pulmonary mechanics by spectral analysis of forced random noise. J. Clin. Investig..

[28] Goldman M.D. (2001). Clinical Application of Forced Oscillation. Pulm. Pharmacol. Ther..

[29] Kaczka D.W., Dellaca R.L. (2011). Oscillation Mechanics of the Respiratory System: Applications to Lung Disease. Crit. Rev. Biomed. Eng..

